# Prior exposure to weathered oil influences foraging of an ecologically important saltmarsh resident fish

**DOI:** 10.7717/peerj.12593

**Published:** 2022-01-05

**Authors:** Ashley M. McDonald, Charles W. Martin, Guillaume Rieucau, Brian J. Roberts

**Affiliations:** 1 UF|IFAS Nature Coast Biological Station, University of Florida, Cedar Key, Florida, United States; 2 Louisiana Universities Marine Consortium, Chauvin, Louisiana, United States

**Keywords:** Fish behavior, Macondo, Nekton, Feeding effort, Mesocosm

## Abstract

Estuarine ecosystem balance typically relies on strong food web interconnectedness dependent on a relatively low number of resident taxa, presenting a potential ecological vulnerability to extreme ecosystem disturbances. Following the *Deepwater Horizon* (DwH) oil spill disaster of the northern Gulf of Mexico (USA), numerous ecotoxicological studies showed severe species-level impacts of oil exposure on estuarine fish and invertebrates, yet post-spill surveys found little evidence for severe impacts to coastal populations, communities, or food webs. The acknowledgement that several confounding factors may have limited researchers’ abilities to detect negative ecosystem-level impacts following the DwH spill drives the need for direct testing of weathered oil exposure effects on estuarine residents with high trophic connectivity. Here, we describe an experiment that examined the influence of previous exposure to four weathered oil concentrations (control: 0.0 L oil m^−2^; low: 0.1 L oil m^−2^; moderate: 0.5–1 L oil m^−2^; high: 3.0 L oil m^−2^) on foraging rates of the ecologically important Gulf killifish (*Fundulus grandis*). Following exposure in oiled saltmarsh mesocosms, killifish were allowed to forage on grass shrimp (*Palaeomonetes pugio*) for up to 21 h. We found that previous exposure to the high oil treatment reduced killifish foraging rate by ~37% on average, compared with no oil control treatment. Previous exposure to the moderate oil treatment showed highly variable foraging rate responses, while low exposure treatment was similar to unexposed responses. Declining foraging rate responses to previous high weathered oil exposure suggests potential oil spill influence on energy transfer between saltmarsh and off-marsh systems. Additionally, foraging rate variability at the moderate level highlights the large degree of intraspecific variability for this sublethal response and indicates this concentration represents a potential threshold of oil exposure influence on killifish foraging. We also found that consumption of gravid *vs* non-gravid shrimp was not independent of prior oil exposure concentration, as high oil exposure treatment killifish consumed ~3× more gravid shrimp than expected. Our study findings highlight the sublethal effects of prior oil exposure on foraging abilities of ecologically valuable Gulf killifish at realistic oil exposure levels, suggesting that important trophic transfers of energy to off-marsh systems may have been impacted, at least in the short-term, by shoreline oiling at highly localized scales. This study provides support for further experimental testing of oil exposure effects on sublethal behavioral impacts of ecologically important estuarine species, due to the likelihood that some ecological ramifications of DwH on saltmarshes likely went undetected.

## Introduction

Trophic connectivity is of vital importance to the functionality and resilience of estuarine ecosystems (Costanza, Kemp & Boynton, 1993; Tett et al., 2013). Greater trophic connections optimize the transfer of primary production to upper trophic levels, increasing food web resilience and maintaining a domain of attraction towards ecosystem stability (MacArthur, 1955; Holling, 1973). Estuarine ecosystems have unique food webs because of the physiological flexibility that is required for resident species to deal with unpredictable abiotic extremes, combined with the limited geologic lifetimes of estuaries, leading to taxonomic diversity limitations (Whitfield, 1994; Day et al., 1989). Therefore, trophic interconnectedness between resident and transient species, and thus food web resilience, of estuarine ecosystems can often rely on a relatively small number of mid-trophic level nektonic estuarine residents (Subrahmanyam & Drake, 1975).

A supported paradigm of estuarine ecosystems is that they are comprised of relatively resilient biota (Elliott & Whitfield, 2011), however severe environmental disturbances may cause disruptions in trophic connectivity (Matich, Moore & Plumlee, 2020). This can stem from negative impacts to “critically resilient” species, described as a taxon with generally high perturbation resilience and an important role in the food web (McCann et al., 2017). In northern Gulf of Mexico (nGoM) estuarine saltmarshes, Gulf killifish (*Fundulus grandis*, Baird and Girard 1853) are a common, opportunistic saltmarsh resident species (Able et al., 2015) with high site fidelity that makes them valuable sentinel species and indicators of habitat disturbance (Nelson, Sutton & DeVries, 2014; Vastano et al., 2017; Jensen et al., 2019). This Cyprinodontiform species is considered highly important to marsh food webs, serving a key function in the “trophic relay” of marsh production to off-marsh open waters *via* predator/prey interactions with larger transient predators (Rozas & LaSalle, 1990; Kneib, 2000; McCann et al., 2017). The saltmarsh habitats of Gulf killifish are characterized by highly dynamic, and often extreme, environmental conditions (*e.g*., extreme temperatures, wide salinity ranges, tidal exchange, hypoxia, *etc*. (Vernberg, 1993)), and are therefore naturally resilient to many interacting stressors, marking Gulf killifish as a “critically resilient” saltmarsh species (Able et al., 2015; Vastano et al., 2017; McCann et al., 2017).

The necessity of understanding how a large-scale disturbance event might disrupt a food web at the critically resilient species level arose with the *Deepwater Horizon* (DwH) oil spill, the largest industrial marine oil spill in American history that impacted nGoM coastlines from Louisiana to Florida (BP Oil Spill Commission, 2011). In 2010, at least 2,113 km of shoreline were subjected to oiling after the explosion of an offshore oil drilling rig on April 20^th^ led to the loss of 11 lives and the corresponding oil well blowout caused the subsequent release of ~4.1 M barrels (~560,000 metric tons) of crude oil into offshore waters (McNutt et al., 2012; Nixon et al., 2016). As the crude oil drifted into coastal areas, where wetlands accounted for ~53% of oiled shoreline (Michel et al., 2013), exposure to the weathered oil caused significant casualties of several critically sensitive species (*sensu* McCann et al., 2017) at low and high trophic levels, such that terrestrial marsh plants, gastropods, predatory birds, and dolphins experienced high levels of mortality. Alternatively, many coastal fish populations and community structures have shown resilience to, or rapid recovery from, the large-scale exposure to weathered oil (Moody, Cebrian & Heck, 2013; Fodrie et al., 2014; Able et al., 2015; Schaefer, Frazier & Barr, 2016; Martin et al., 2020a). A decade later, potential trophic implications from this marine disaster are still unclear, despite extensive analyses of community-wide data, with interpretation of oil spill effects on populations of estuarine and marine species further complicated by possible trophic release (*e.g*., stark decline of mammal and bird predators), engineered efforts to protect shorelines from oil intrusion (*e.g*., boom deployment and increased freshwater discharge), and the extensive temporary closure of Gulf of Mexico fisheries operations (Upton, 2011; Fodrie et al., 2014; McCann et al., 2017). Large gaps in understanding possible trophic repercussions of a marsh community to oil spill effects are therefore bolstered by experimental manipulations not constrained by the unethical re-creation of oiled field conditions, but are instead replicated in a hyper-realistic, controlled mesocosm environment.

Here, we describe the results of a foraging experiment carried out to test the hypothesis that prior oil exposure negatively affects natural predatory feeding rates of Gulf killifish, a dominant and ecologically important marsh resident (Able et al., 2015). Classic foraging experiments examining saltmarsh predator-prey interactions have used killifish (*Fundulus* sp.) and grass shrimp (*Palaeomonetes pugio*) as study species because of their abundance and trophic importance in Atlantic and Gulf coastal marshes (Heck & Thoman, 1981; Kneib, 1987; Kneib, 1988), and because grass shrimp are a common prey item for Gulf killifish of the size range used in this experiment (Harrington & Harrington, 1961). We therefore examined foraging rates of Gulf killifish on unexposed grass shrimp prey, following exposure to varying real-world DwH concentrations of weathered oil in large marsh mesocosms, to assess previous oil exposure impacts explicitly due to exposure of this important saltmarsh resident predator. We tested the hypothesis that foraging rate would decline concomitantly with increased levels of exposure concentration. This investigation was inspired by field reports of little evidence for Gulf killifish population-level effect following nGoM saltmarsh oiling (Able et al., 2015), despite strong negative individual-level physiological and developmental impacts from oil exposure (Garcia et al., 2012; Whitehead et al., 2012; Dubansky et al., 2013; Crowe et al., 2014; Fodrie et al., 2014). Ecotoxicological studies on fish have shown strong evidence that shifts in feeding behavior by sublethal exposure to toxic contaminants may impact community structure or trophic transfer caused in part by decreased motivation to feed, impaired feeding abilities, and decreased prey detection (Atchison, Henry & Sandheinrich, 1987; Little et al., 1990; Weis et al., 2001; Fleeger, 2020). Decreased Gulf killifish foraging rates related to any previous sublethal oil exposure would provide further evidence that this type of environmental disturbance could influence nearshore food web connectivity.

## Materials and Methods

### Oiled mesocosms and killifish exposure

During August/September 2019, an ongoing experimentally oiled marsh mesocosm experiment was being conducted at Louisiana Universities Marine Consortium (LUMCON) in Cocodrie, LA (29.254573°, −90.664031°). These oiled mesocosms were used to expose Gulf killifish for 10-15 days, a duration based on previous studies of site fidelity for *F. grandis* (Nelson, Sutton & DeVries, 2014; Jensen et al., 2019). Twelve hydrologically-independent, outdoor mesocosms (3.05 m diameter, 1.83 m height) were paired each with its own tidal surge tank that generates daily tidal cycles with range of 25 cm (flooding marsh ~10 cm at high tide) *via* a water control system of blowers and airlifts (Alt, 2019). Intact *Spartina alterniflora* saltmarsh plugs (30 cm diameter × 50 cm depth) at natural densities were collected from previously unoiled natural saltmarshes near the LUMCON mesocosm facility, planted in the mesocosms, and allowed to establish for approximately 18 months prior to oiling (Roberts et al., 2019; Roberts et al., 2020). During flooded marsh conditions (~40% of the time), Gulf killifish had access to ~7.3 m^2^ of marsh platform (~10 cm at high tide) to allow for natural foraging opportunities, and adjacent deeper water (~40 cm at high tide) in the empty circular trough surrounding the saltmarsh platform. At low tide, Gulf killifish were restricted to the ~1.4 m^2^ of area in the trough perimeter, with minimum water depths of 15 cm at low tide. Additional details on the saltmarsh mesocosm setup and design can be found in Fig. S1.

Oil used in mesocosm exposure periods was Light Louisiana Sweet (LLS) blended crude oil at API Gravity 40.1, similar to the oil released by the DwH spill, acquired from Placid Refining Company LLC in May 2018. The oil was then evaporatively weathered using a nitrogen gas sparging system over 150 days to obtain a loss of 30% of volatile components, as measured by gas chromatography, to attain chemical composition similar to that of weathered oil that washed ashore following DwH (Passow & Overton, 2021). Four exposure levels were randomly assigned to three replicate mesocosms in a randomized block design for a total of 12 experimental mesocosm units. Weathered oil exposure levels scaled roughly to Shoreline Cleanup and Assessment Team (SCAT) categories observed in Louisiana marshes following the DwH disaster (Michel et al., 2013; Lin et al., 2016): control/no oil at 0.0 L oil m^−2^, low at 0.1 L oil m^−2^, moderate at 0.5–1 L oil m^−2^, and high at 3.0 L oil m^−2^. For the oiled treatment mesocosms, a single application of weathered oil was applied to the saturated sediment surface under high tide conditions at uniformly spaced locations on July 8, 2019. After initial weathered oil application, oil was further naturally weathered in the open air of mesocosms 45 to 60 days prior to experiment initiation.

During the killifish exposure period, following 45 to 60 additional days of open-air oil weathering, the mean ± standard error surface soil (0-5 cm) total petroleum hydrocarbon (TPH) concentrations in the high oil treatments (419 ± 24 mg/g soil) were ~10 and ~40 times higher than in the moderate (39 ± 5 mg/g soil) and low (10 ± 0.4 mg/g soil) oil treatments (mean of 19 August and 9 September samplings; as previously described in Martin et al., 2020b, and collected by E. Overton and B. J. Roberts, 2020). These concentrations are similar to those found in Louisiana saltmarsh field conditions following the DwH oil spill (Lin et al., 2016). Gulf killifish were collected from the nearby saltmarsh using baited minnow traps and held for 2–4 days in the same 450 liter, aerated aquarium with a constant salinity of 7 psu (equivalent to mesocosms at the time of collection) to minimize mortality due to handling. Then, 12 adult fish were added to each saltmarsh mesocosm on 22 August 2019, with six more Gulf killifish added to each mesocosm on 27 August to increase the number of fish available for the foraging behavior experiment after some mortality was observed after 5 days of holding in mesocosms. Total fish mortality during the exposure period resulted in no mortality in control treatments, 38% in low exposure treatments, 56.6% in medium exposure treatments, and 81.5% in high exposure treatments, however the causes of mortality were not determined and possibly arose from direct and indirect effects from oil exposure (see Martin et al., 2020b for further detail on fish additions). All surviving fish used in the subsequent foraging experiment (three to six fish per mesocosm) were within the size range of 57 to 105 mm (mean of lengths 79.4 + 1.8 mm standard error). Fish were exposed to oiled or control treatments for 10 (27 August additions) to 15 (22 August additions) days prior to behavioral experiments. Following the exposure period, fish were recaptured with dipnets and kept separated according to mesocosm assignment then held without food for 24 h at ambient room temperature conditions in aerated 37.9 L aquaria containing unoiled 7 psu filtered seawater prior to use in the foraging experiment performed at the LUMCON facility.

### Foraging experiment

Gulf killifish foraging behavior response to previous oil exposure was examined over a one-day period. Foraging trials were conducted at the LUMCON facility in separate 19 L white plastic buckets filled with 10 L of filtered water with a salinity of 7 psu. Twenty grass shrimp (*Palaemonetes pugio*), captured by dipnet from the nearby bayou were added to each bucket and allowed to acclimate for 2 h prior to the start of the experiment. Shrimp densities were selected to represent natural field densities relative to the size of the experimental unit (~67 shrimp per m^2^). As shrimp were added, we noted the number of gravid shrimp in each bucket as a potential foraging covariate due to the possibility that gravid shrimp may be more visually/olfactorily detectable or less capable of escape, making them more easily predated by impaired killifish (Welsh, 1975). However, we did not control for the same proportion of gravid to non-gravid across replicates. To better simulate the low marsh habitat structure where killifish forage most effectively (Vince et al., 1976), ten 18 cm length black needlerush (*Juncus roemerianus*) stems were added to each bucket in a haphazard fashion.

To initiate the experiment, killifish were transferred from the 37.9 L holding aquaria to 19 L buckets containing the grass shrimp that had been randomly assigned a killifish oil exposure or autogenic control treatment. Killifish from the same oiled mesocosm treatment were randomly paired by drawing numbers without replacement, and the two fish placed into one of nine replicates for control, low, or moderate treatment buckets but only five replicates for high treatment buckets, which precluded equivalent replication for all treatments tested in the foraging experiment. No further criteria were used to select killifish inclusion in the experiment. An additional four autogenic control buckets contained only grass shrimp with no killifish addition for a total of 36 buckets arranged in a 6 × 6 grid with rows spaced 1 m apart. Every 4 h during the experiment we monitored factors that would affect foraging rates, specifically mortality of killifish and complete predation of all shrimp provided to a replicate. To minimize investigator influence on foraging, inspections consisted of identifying shrimp and killifish movement from a distance of 0.5 m, and randomization of experimental treatments within the grid array ensured that any observer influence was not grouped. Water temperatures ranged from 26.8–28.6 °C during trials (comparable to mesocosm conditions at time of collection) and dissolved oxygen remained near 100% saturation, determined by measurements at beginning and end of trial. At 21 h, all killifish were removed from the buckets and remaining gravid and nongravid shrimp were counted and these values deducted from gravid and nongravid shrimp counts taken at the beginning of the experiment. To calculate shrimp consumption rate per fish per minute (shrimp consumed fish^−1^ min^−1^), the number of shrimp consumed within each bucket was divided by the sum of foraging minutes for both fish in that bucket. If fish mortality or complete predation was noticed during routine checks, the reduced foraging time for that fish or bucket was noted and values adjusted accordingly. Mortality was recorded during the foraging experiment, with the following losses per oil exposure treatment: control-one, low-none, medium-four (two from the same bucket), and high-one. These losses corresponded to ~6% mortality in the control treatment, 0% mortality in low oil exposure treatment, ~22% mortality in medium oil exposure treatment, and 10% mortality in high oil exposure treatment. All experimental units were included in subsequent analyses.

## Statistical Analyses

Due to the unbalanced sample sizes (*n* = 9 for control, low, moderate prior oil exposure and *n* = 5 for high prior oil exposure) and heteroscedasticity of experimental results, a Welch’s ANOVA was used to compare Gulf killifish foraging rates among weathered crude oil exposure treatments and a Games-Howell test was used for *post hoc* contrasts (Moder, 2010). A linear regression with combined fish lengths per replicate and foraging rate was used to determine whether killifish length should be included as a potential covariate with oil exposure treatment. To test the hypothesis that gravid grass shrimp consumption was independent of prior oil exposure concentrations, we constructed a chi square table on expected and observed gravid shrimp consumption. For this analysis, we excluded any buckets where the 20 shrimp provided for foraging during the experiment did not include gravid individuals (excluded one control, three moderate, and one high oil exposure treatment replicates). To estimate how many gravid shrimp we would expect to be eaten with the assumption killifish had no preference, we multiplied the proportion of gravid shrimp given to a treatment by the total number of shrimp eaten, noting that both values excluded nongravid shrimp counts from buckets that did not receive gravid shrimp. For example, if 10% of shrimp provided to a treatment were gravid and 50 shrimp were consumed by that treatment group, our expected value for that treatment would be five gravid shrimp consumed. Analyses were performed in RStudio (Version 1.2.1335) and the accepted α was set at 0.05 for the Welch’s ANOVA, Games-Howell contrasts, linear regression, and chi square test.

### Vertebrate study animal ethics statement

Gulf killifish were collected from marsh sites adjacent to the LUMCON facility in Chauvin, LA. All field collections were made under Louisiana Department of Wildlife and Fisheries Scientific Collecting Permit #SCP 200. The use of vertebrate organisms was conducted with IACUC approval and staff training from University of Florida under protocol 201710044. Fish were held in 3.05 m diameter simulated marsh mesocosms during the weathered oil exposure period, where they were allowed to freely forage under simulated natural bayou conditions. As the goal of the study was to measure sublethal effects of oil on fish behavior, humane endpoints were not used and were not possible during the 10–15 day exposure, as fish were released into turbid mesocosms and unable to be monitored. Moreover, analgesics and anesthetics were not used because of the alterations to behavior that we sought to quantify. During the one-day experiment, fish were held in 19 L plastic buckets filled with 10 L of filtered water with salinity of 7 psu. At the end of the experiment, surviving fish were euthanized humanely using the standard methodology for finfish as outlined in IACUC protocol 201710044-namely, through cold shock immersion in ice slurry followed by immersion in 500 mg/L MS-222 (buffered tricaine methane sulfonate) for 10 min after opercular movement stops.

## Results

**Figure 1:**
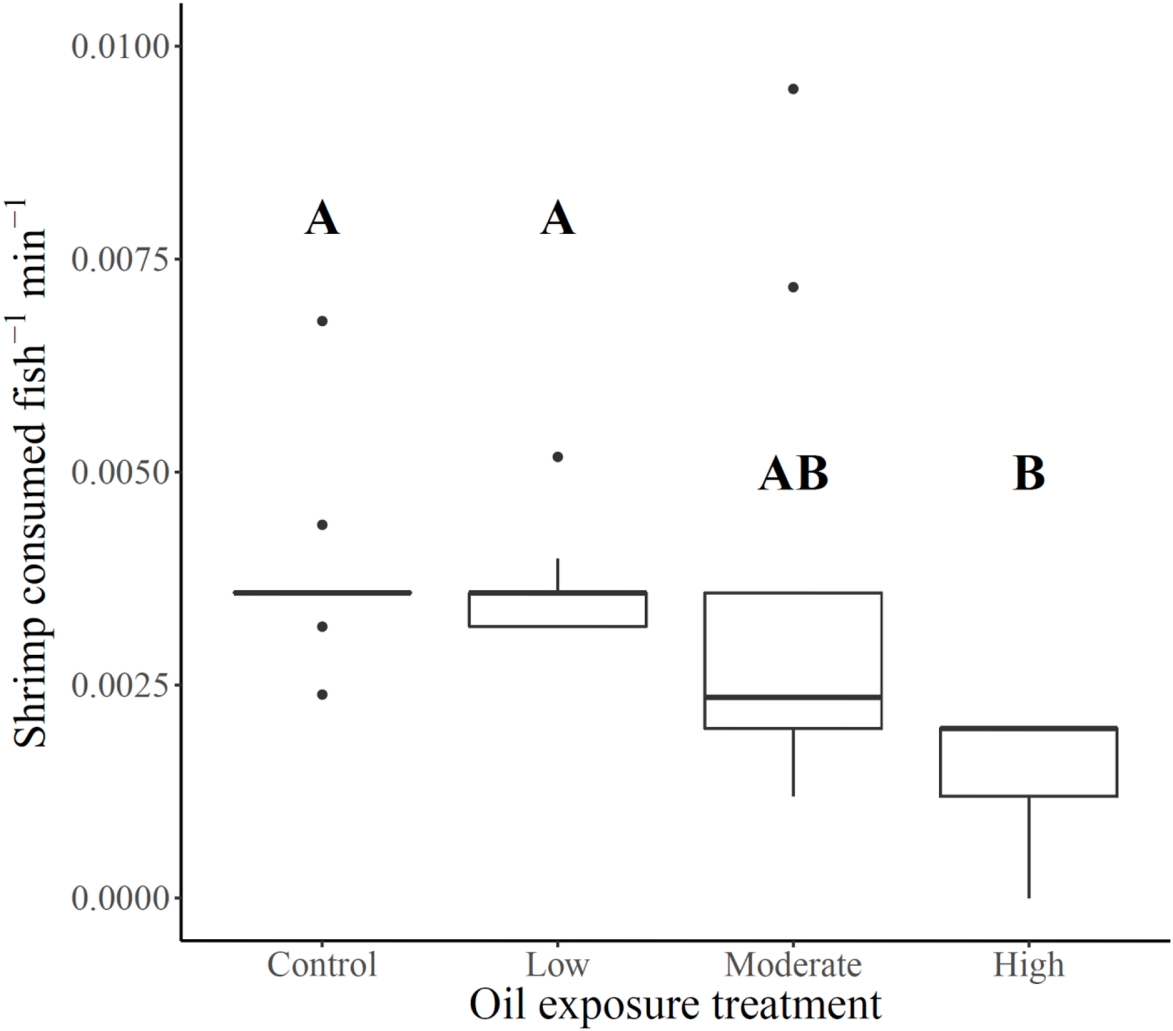
Gulf killifish foraging rates per replicate for each prior oil exposure treatment. Gulf killifish foraging rates (number of shrimp consumed fish^−1^ min^−1^) per bucket replicate for each prior oil exposure treatment. Solid lines in boxes represent treatment medians and dots are outliers. Different letters indicate treatment differences based on results from Games-Howell post-hoc contrast.

Shrimp consumption ranged from 18% of shrimp offered to high exposure killifish to 48% of shrimp offered to unexposed control killifish, with low and moderate exposure killifish exhibiting slightly lower overall consumption than control killifish (46% and 42%, respectively). Foraging rates (shrimp consumed fish^−1^ min^−1^) varied across oil exposure treatments (Fs_3,12.9_ = 8.2, *p* = 0.0026), such that rates for killifish from high oil exposure treatments were lower than for killifish from the low and control treatments (Fig. 1 and Table 1). Although average foraging rates of moderate oil exposure killifish were between the averages for low and high exposure treatments, moderate treatment rates showed substantial within-treatment variability (Table 1) that included the two highest foraging rates of the experiment (0.01 and 0.007 shrimp consumed fish^−1^ min^−1^) as well as four measurements of low rates (ranges between 0.001–0.002 shrimp consumed fish^−1^ min^−1^) comparable only with high exposure treatment fish (all rates ranged from 0–0.002 shrimp consumed fish^−1^ min^−1^). No relationship was found between combined fish length per replicate and foraging rate (linear regression: Adj. r^2^ = 0.002, F_1,30_ = 1.07, *p* = 0.31), therefore fish length was not considered as a potential covariate.

**Table 1:** Basic statistics for Gulf killifish foraging rates at prior oil exposure treatment concentrations.

Oil addition treatment	*N*	Experiment mortality (number of fish)	Mean foraging rate per bucket (shrimp min^−1^)	Standard deviation	Coefficient of variation
Control (0.0 L m^−2^)	9	1	0.003851	0.00121	31.4
Low (0.1 L m^−2^)	9	0	0.00363	0.00064	17.6
Medium (0.5–1.0 L m^−2^)	9	4	0.00359	0.00283	78.8
High (3.0 L m^−2^)	5	1	0.001434	0.00087	60.7

**Figure 2:**
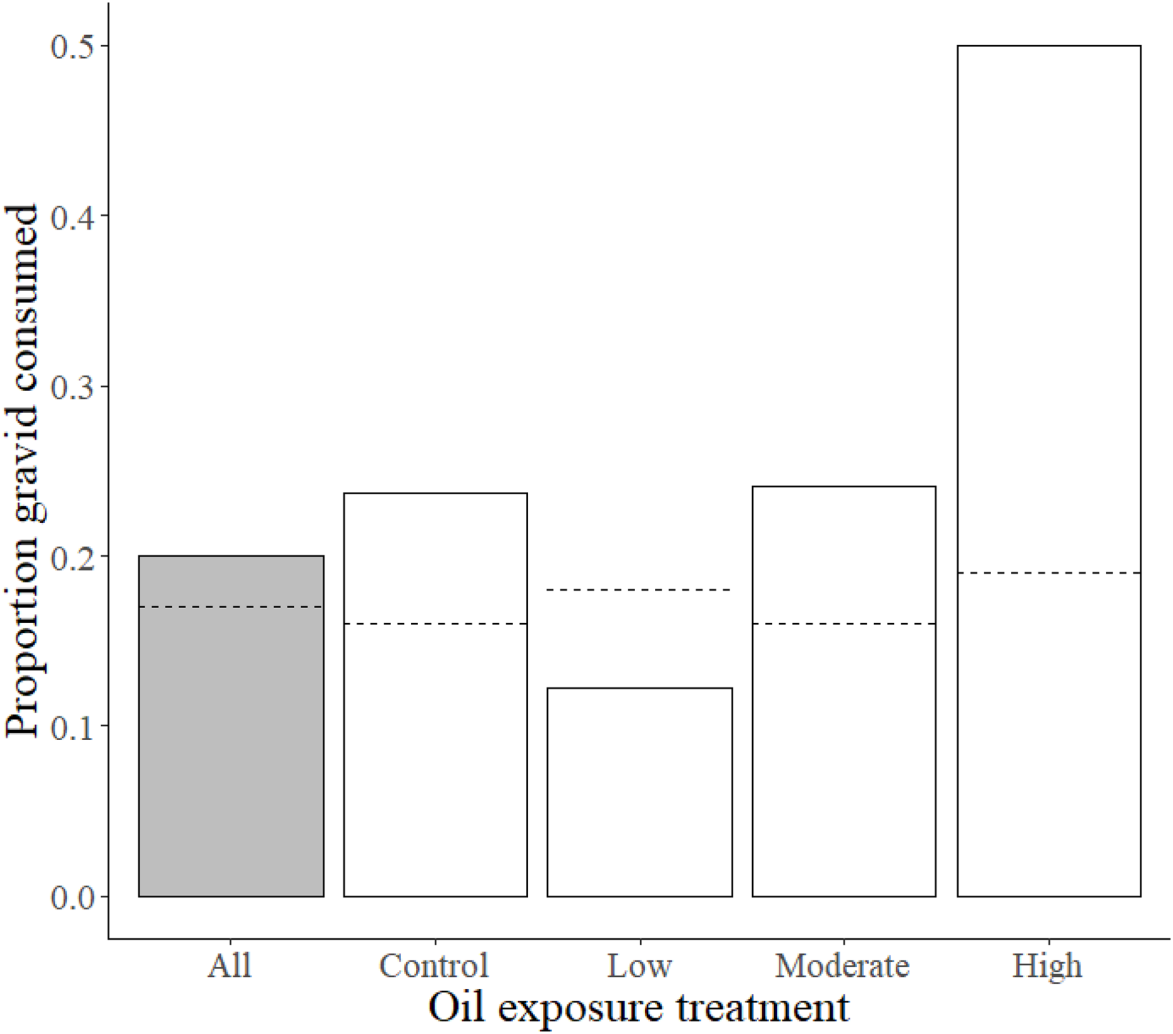
Proportions of gravid shrimp consumption by Gulf killifish. Proportions of observed values of gravid shrimp consumed (out of total shrimp consumed) by Gulf killifish for the entire experiment (grey bar) and at different treatment levels of prior oil exposure, with the calculated expected proportion values represented by dotted lines.

Previous exposure to the high oil concentration influenced preference for gravid over non-gravid shrimp, such that high exposure fish consumed a substantially larger proportion of gravid shrimp (0.5) than moderate, low, and control treatment killifish (0.24, 0.12, and 0.24, respectively). This observed proportion of gravid shrimp consumed in high oil treatment fish deviated from the expected high exposure consumption proportion of 0.19 (χ^2^ = 10.1, df = 3, *p* = 0.017; Fig. 2 and Table 2).

**Table 2:** Values to calculate expected *vs* observed outcomes of gravid shrimp consumption for construction of chi square table.

Oil addition treatment	Total shrimp consumed	Proportion of gravid	Expected gravid consumed	Observed gravid consumed
Control (0.0 L m^−2^)	76	0.16	11.8	18
Low (0.1 L m^−2^)	82	0.18	14.8	10
Medium (0.5–1.0 L m^−2^)	29	0.16	4.7	7
High (3.0 L m^−2^)	8	0.19	1.5	4

## Discussion

Our study provides evidence that prior exposure to real-world concentrations of weathered oil (Lin et al., 2016) influences foraging of estuarine saltmarsh resident Gulf killifish. Our results reveal a substantial reduction of killifish foraging rates at high oil exposure concentration, a highly variable effect in the moderate concentration, and an absence of effect in the low concentration exposure. Our finding that low and moderate concentration exposures did not substantially influence foraging rate is counter to prior experimental evidence for killifish behavioral changes at even the lowest previous oil exposure concentration (Martin et al., 2020b), and strongly suggest that a threshold of sublethal effects on foraging, and possibly individual-level response plasticity (Saaristo et al., 2018), is evident around the crude oil concentrations in the moderate exposure treatment. Our experimental design to test the influence of prior oil exposure on foraging rates was challenged by a reduction in sample sizes caused by mortality of subjects during the exposure period, particularly in the high exposure treatment. However, the foraging behavior responses of surviving killifish from high oil concentration mesocosms were consistently affected by this exposure, providing strong evidence that these sublethal effects are genuine. We also found evidence that fish from the high exposure treatment consumed gravid grass shrimp in a higher proportion than expected, while fish from all other treatments did not exhibit this selectivity, novel evidence of a contamination exposure-related shift in killifish predatory behavior with potential for ecological repercussions (see Saaristo et al., 2018 Table S2 for compilation of studies on contaminant effects on predator-prey interactions). These findings indicate prior oil exposure can influence killifish foraging rates and may drive a behavioral shift in prey selection, at least on the spatial and temporal scales used in this study. Despite little field-based evidence of large-scale negative community impacts (Fodrie et al., 2014; Martin et al., 2020a), the consistent evidence of negative physiological, genomic, and developmental effects on nekton from oil exposure strongly suggests there are key gaps in our understanding of coastal trophic processes that were likely disrupted in part by sublethal effects on nearshore nekton behavior. Our study findings support this inference, as high oil exposure substantially affected Gulf killifish foraging behavior, and moderate oil exposure highlighted the high degree of intraspecific variability for sublethal responses to oil at the thresholds tested.

High inter-individual variability in the moderate treatment foraging rates reveals the potential presence of phenotypic plasticity within this subsample of a local killifish population in response to moderate levels of contamination, a vital feature for enabling species to cope with rapid environmental change (Chevin, Lande & Mace, 2010). Yet despite the exceptionally high foraging rates of some individuals, the overall average foraging rate for this treatment was still lower than control and low concentration treatments, suggesting population-level effects on foraging rates at this concentration may have occurred despite high individual-level plasticity. A potential behavioral shift influence on intra-population dynamics relates to our findings that high oil concentration exposure fish selected gravid shrimp at higher proportions than expected if shrimp selection were independent of prior oil exposure. We speculate that this observation could be related to an impairment in prey detection (Cave & Kajiura, 2018), as gravid grass shrimp are more visually apparent than non-gravid grass shrimp. Secondly, gravid selection may relate to killifish mobility impairment (Stieglitz et al., 2016), as killifish are active predators and gravid grass shrimp may have reduced escape capabilities since swimmerets are occupied with egg masses. However, evidence of the relationship between prior high oil exposure and increased killifish selection of gravid grass shrimp presented here is a preliminary finding due to the limited scale of our foraging experiment design, the potential size difference between gravid and male shrimp (Alon & Stancyk, 1982), and the fact that we only exposed the predatory killifish, and not the prey species, to weathered oil.

The reduced predation success of Gulf killifish previously exposed to high concentration of weathered oil measured in this study reflects findings of reduced growth and predation of an Atlantic congeneric, mummichog (*Fundulus heteroclitus*), inhabiting contaminated estuaries (Weis et al., 2001). Killifish from contaminated estuaries were found to rely primarily on low nutritional value detrital material for forage, as opposed to higher calorie prey items that constitute a greater dietary proportion in fish from an uncontaminated site (Weis & Khan, 1991). Previous foraging experiments suggested contaminated site fish generally could not capture evasive grass shrimp prey, and maintained low activity levels (Smith et al., 1995). Grass shrimp are highly abundant and productive estuarine saltmarsh residents (Nixon & Oviatt, 1973) that effectively transfer detritus into higher saltmarsh trophic levels, particularly large killifish, through predator-prey interactions (Nixon & Oviatt, 1973; Welsh, 1975). The sublethal effect of prior exposure to high concentrations of oil on foraging rates may result in a similar influence on the killifish foraging guild as found in contaminated estuaries (Weis et al., 2011). Namely, a depression of predatory foraging wherein killifish diets become dominated by detritus rather than energetically superior grass shrimp which drives a declining efficiency in the transfer of saltmarsh-originated energy to off-marsh systems.

Although our study was not designed to investigate the physiological or genomic impairments that may have caused changes in Gulf killifish foraging behavior due to short term oil exposure, evidence from previous oil and fossil fuel toxicity-related studies on fish suggest numerous physiological impairments that can impact foraging abilities (Gregg, Fleeger & Carman, 1997; Stieglitz et al., 2016; Cave & Kajiura, 2018; Schlenker et al., 2019). Field studies that examined adult Gulf killifish genome expression during the oil spill aftermath (Whitehead et al., 2012; Dubansky et al., 2013) found evidence for damage of gill tissues, an organ that is vital to many functions necessary for physiological resilience of an estuary resident organism. The ecotoxicological effects from exposure to petroleum hydrocarbons can also be compounded by natural abiotic stressors common to estuarine environments, such that energy demands required for fish to compensate for natural stressors are extra costly (Whitehead, 2013). Time spent in natural estuarine conditions within various weathered oil exposures of the mesocosms likely impacted energetics budgets of fish used in our experiment, possibly contributing to the pattern of mesocosm mortality and foraging inhibitions during the experiment. Furthermore, evidence of oil ingestion in Gulf killifish four to 5 months after the cessation of the oil spill (Dubansky et al., 2017) is a potential pathway for the extended oil exposure influence on these fish linked to their preference for benthic-associated forage (Rozas & LaSalle, 1990), as the more viscous weathered oil settles into sediments and remains in the environment longer due to anoxic conditions (Mendelssohn et al., 2012).

Despite the noted resilience of this species (Able et al., 2015), it is possible that any sublethal impacts driving these behavioral shifts on trophic transfer or food web resilience were highly localized and thus went undetected. Natural avoidance behaviors to oil have been recorded in several fish species (Martin, 2017) that in the wild may have somewhat alleviated the more extreme foraging inhibition we measured in high oil exposure treatment fish. However, these avoidance behaviors have been found to degrade over sediments contaminated with weathered oil, as opposed to fresh oil (Martin, 2017), and upon the fish having prior exposure to weathered oil at even low levels (0.1 L oil m^−2^), with some evidence of preference for oiled sediments following exposure to high levels (3.0 L oil m^−2^) (Martin et al., 2020b). The various levels of oiling were patchily distributed across nGoM shorelines such that surveys found ~20% of oiled shorelines were heavily oiled, ~13% were moderately oiled, and ~36% were lightly oiled (Michel et al., 2013). These surveys further determined ~53% of total oiled shoreline was marsh habitat, and of that, ~96% of oiled marsh habitat was in Louisiana estuarine environments (Nixon et al., 2016). The patchy distribution of oil on diverse saltmarsh subhabitats characterized by spatially heterogenous estuarine saltmarsh nekton communities (Peterson & Turner, 1994; Able et al., 2015) that tend to exhibit high site fidelity (Nelson, Sutton & DeVries, 2014; Jensen et al., 2019) suggests that if oil exposure negatively impacted Gulf killifish foraging to the degree that it impacted trophic connectivity, this impact would have been highly spatially variable. High intraspecific redundancy of Gulf killifish combined with high individual-level variability in foraging response to oil exposure, such as that measured in our moderate oil treatment, may have worked to lessen oil exposure impacts on this estuarine nekton species’ contributions to saltmarsh food web connectivity.

Our study highlights the need for more experimental and mesocosm-based work to aid with further determinations of what additional sublethal impacts likely occurred in the aftermath of the DwH oil spill, and to help predict how toxic stressors may influence trophic processes in these critical coastal habitats. The extreme variability in killifish foraging responses to moderate oiling suggests that thresholds to negative sublethal effects from oil exposure are highly individualized, but perhaps lower thresholds are within this concentration range, at least at the timeframe of exposure of this experiment. This is potentially the result of intraspecific variability in tolerance to the general “narcosis” effect of unspecific baseline toxicity to organic narcotic compounds (Hsieh, Tsai & Chen, 2006), generally considered a reversable process that causes a variety of responses dependent on the exposure timeframe and affecting compound (Heintz, Short & Rice, 1999; Vines et al., 2000). Additional experimentation at this moderate exposure concentration may provide more insight into whether foraging declines in this treatment were, in fact, a reversible response to the narcosis effect or if high foraging rates may reflect individuals with higher tolerance to this effect. While large-scale resilience of nGoM estuarine saltmarshes to oiling is apparent, continued release of buried oil through marsh erosion and re-oiling of marshes following large storm events may redistribute oil or oil residues for decades (Turner et al., 2019). Thus, sublethal nekton responses to oil exposure may continue to influence trophic connectivity and food web resilience at highly localized scales- making the detection of eroding food web resilience extremely difficult. However, the hearty resilience of a common and dominant nGoM estuarine saltmarsh resident with high intraspecific variability up to the moderate levels of oiling tested here suggests that this near-shore ecosystem has some resilience built into the food web, providing some stability despite a major ecotoxicological perturbation.

## Conclusions

To supplement numerous field sampling studies examining oil spill impacts on GoM shoreline habitats, we tested the hypothesis that prior oil exposure influences foraging behavior of Gulf killifish by conducting feeding trials following exposure to varying levels of oil within an ongoing mesocosm experiment. Our experimental results indicate that previous exposure to weathered oil at moderate to high levels can have highly variable to substantially negative impacts on foraging behavior of this critically resilient saltmarsh resident. The high variability at moderate oiling level, as opposed to no effect at control and low levels and negative effect at high level, suggests this concentration represents a threshold of oil exposure influence on Gulf killifish foraging. High exposure treatment fish exhibited more selective predation behavior than other exposure treatments by consuming a higher proportion of gravid than expected, suggesting sublethal impacts to foraging abilities. Inefficient predation by killifish has been suggested to directly influence size-frequency distributions of grass shrimp (Bass et al., 2001), and therefore this preliminary finding warrants further investigations into the potential selective foraging behaviors of Gulf killifish exposed to weathered oil and how these behaviors may impact local population dynamics of both predator and prey species. Although our findings are limited in scope and based on results of one experimental trial, any discovery of potential sublethal oil exposure effects on organismal behavior is inherently vital. Based on these results, we speculate that trophic transfer of energy from the marsh platform to off-marsh and open water systems may have been impacted by weathered oil, albeit heterogeneously across saltmarsh habitat and on a highly localized scale. Further experimental studies examining oil exposure on ecologically valuable species is warranted to better determine behavioral implications from previous and potential future environmental disasters.

## Supplemental Information

10.7717/peerj.12593/supp-1 Supplemental Information 1 Raw dataset.

10.7717/peerj.12593/supp-2 Supplemental Information 2 Oiled mesocosm layout and close-up. (A) The salt marsh mesocosm system is made of 12 hydrologically independent tanks (10’ diameter, 5’ tall) with paired tidal surge tanks (6’ diameter) capable of generating tidal ranges up to 60 cm. (B) Each mesocosm tank consists of (from bottom to top) a gravel layer with a French drain, geocloth, sand, soil collected from nearby natural salt marsh channels, and a top layer of marsh plants/roots/soil that is dominated by *Spartina alterniflora*. To minimize variability between tanks, we planted a uniform community of salt marsh grass collected as intact sections from the nearby natural marsh.

## Additional Information and Declarations

### Field Study Permissions

The following information was supplied relating to field study approvals (*i.e*., approving body and any reference numbers): All field collections were made under Louisiana Department of Wildlife and Fisheries Scientific Collecting Permit #SCP 200.

### Data Availability

The following information was supplied regarding data availability: The data are publicly available through the Gulf of Mexico Research Initiative Information & Data Cooperative (GRIIDC): Martin, C. W., Roberts, B. J., 2020. Laboratory experiments to determine effects of previous exposure to oil on *Fundulus grandis* avoidance behavior. Distributed by: Gulf of Mexico Research Initiative Information and Data Cooperative (GRIIDC), Harte Research Institute, Texas A&M University–*Corpus* Christi. doi 10.7266/n7-3qjh-2f11.
