# Supplementary material for: Prior exposure to weathered oil influences foraging of an ecologically important saltmarsh resident fish
Source: PeerJ. 2022 Jan 5;10:e12593. doi: 10.7717/peerj.12593 (PMC8742545; doi:10.7717/peerj.12593)
Supplement: Supplemental Information 2 — (A) The salt marsh mesocosm system is made of 12 hydrologically independent tanks (10’ diameter, 5’ tall) with paired tidal surge tanks (6’ diameter) capable of generating tidal ranges up to 60 cm. (B) Each mesocosm tank consists of (from bottom to top) a gravel layer with a French drain, geocloth, sand, soil collected from nearby natural salt marsh channels, and a top layer of marsh plants/roots/soil that is dominated by Spartina alterniflora. To minimize variability between tanks, we planted a uniform community of salt marsh grass collected as intact sections from the nearby natural marsh. [file peerj-10-12593-s002.pdf]

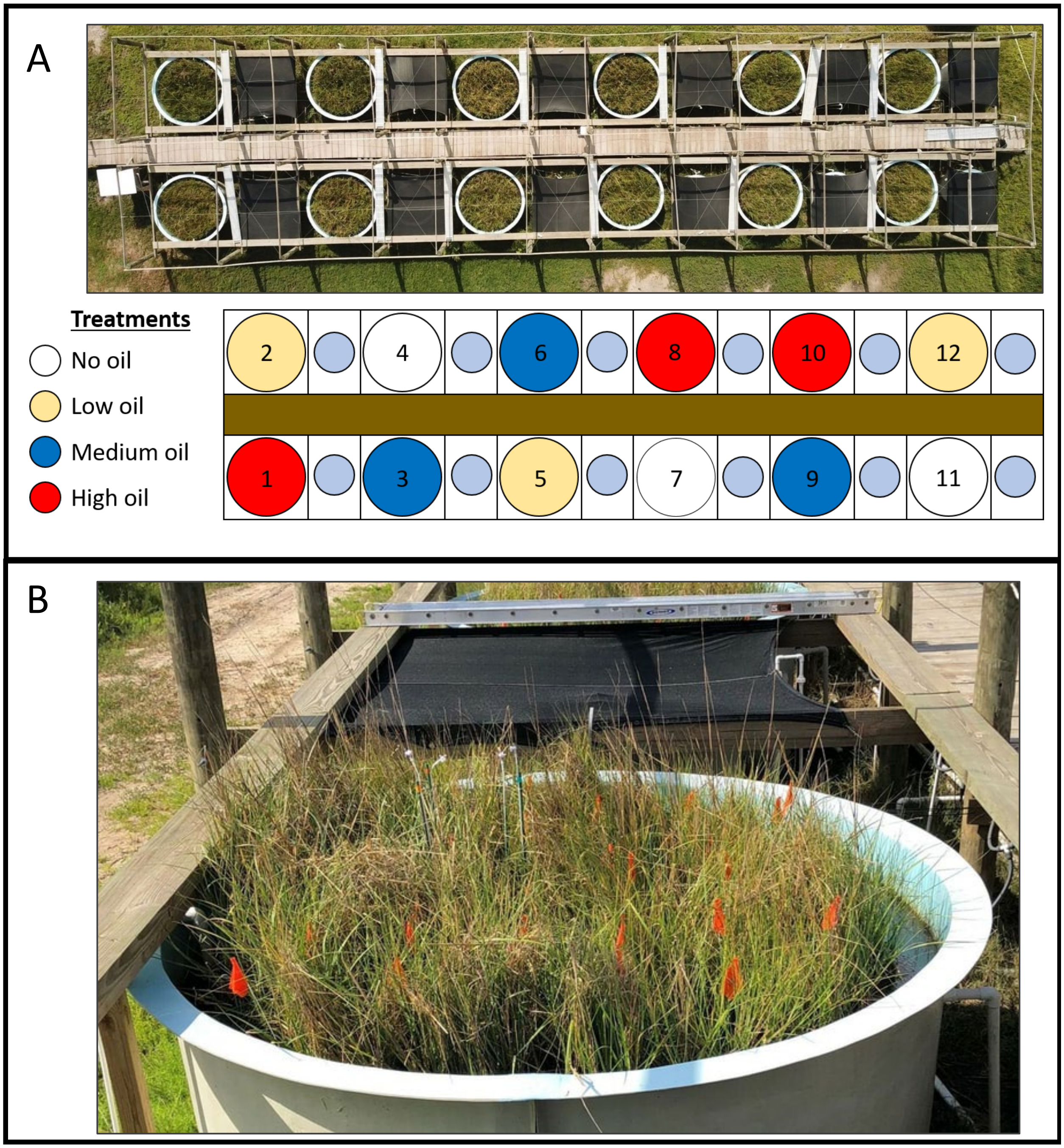
A
Treatments
No oil
Low oil
Medium oil
High oil
2
4
6
8
10
12
1
3
5
7
9
11
B
